# Generation paths of major production safety accidents — A fuzzy-set qualitative comparative analysis based on Chinese data

**DOI:** 10.3389/fpubh.2023.1136640

**Published:** 2023-03-23

**Authors:** Fujun Zhou, Jian Zhang, Cheng Fu

**Affiliations:** ^1^School of Public Administration, Central South University, Changsha, Hunan, China; ^2^Research Centre for Rural Revitalization of Central South University, Changsha, China

**Keywords:** safety management, major production safety accidents, causality, generation mechanism, QCA

## Abstract

Major production safety accidents have become the key obstacle to improve the overall safety production level. Analyzing the causal relationship of major production safety accidents is helpful to clarify its characteristics and laws. Based on the literature, the analytical framework of “individual - technology - management - environment” is proposed. Taking 37 production safety accidents as samples, fuzzy set qualitative comparative analysis (fsQCA) is used to analyze the occurrence path and mechanism of major production safety accidents. The results show that: (1) Major production safety accidents are the result of multiple factors coupling. Minor external supervision or abnormal production behaviors are more likely to cause major production safety accidents. (2) When the production behavior is abnormal and the safety management ability is insufficient, major production safety accidents are more likely to occur. (3) There are five ways and three mechanisms for major production safety accidents. This work enriches the cognition of causality of production safety accidents from the perspective of configuration, clearly shows which variable combinations lead to major accidents, and helps to prevent and reduce major production safety accidents and their risks.

## 1. Introduction

Although a large amount of manpower, material and financial resources have been invested, while still production safety accidents are the main reason threatening safety in production. From 1949 to 2009, there were 26 major coal mine production accidents with more than 100 deaths in China (1). It has a negative impact on economic development, social stability and personal life safety. It is worth noting that major production safety accidents are regarded as an important safety management issue worldwide. Compared with ordinary accidents (less than 3 deaths, or less than 10 serious injuries, or less than 10 million yuan of direct economic loss), major accidents (more than 10 and less than 30 deaths, or more than 50 and less than 100 were seriously injured, or more than 50 million yuan and less than 100 million yuan of direct economic loss), or extraordinarily major accidents (more than 30 deaths, or more than 100 serious injuries, or more than 100 million yuan of direct economic loss) (2) are the main obstacles to improving the overall production safety. Major production safety accidents have serious consequences and high frequency. Taking China as an example, 34,600 production safety accidents and 26,300 deaths occurred in 2021 (3). In addition, the management aiming at reducing major production safety accidents has not reached the ideal state. What causes major production safety accidents deserves further consideration. Through the analysis of the development process of production safety accidents, we can better understand the causes and paths of major production safety accidents, and explore the governance mechanism of major production safety accidents. Therefore, it is urgent and challenging to study and analyze major production safety accidents.

There are a lot of literatures explaining the occurrence and causes of production safety accidents, revealing the essential laws of production safety accidents. The main causes of production safety accidents are as follows.

Individual factors. According to the research of accident prevention benefit analysis model (4), the main cause of production safety accidents is human factors. Some studies have carried out statistical analysis of various production safety accidents from 2001 to 2011. The results show that the accident rate caused by human factors is about 88% (5). Personal factors leading to production safety accidents include unsafe behaviors (6) and worker characteristics (7). First, production behavior is closely related to work experience, skills and knowledge, age and other factors. The structural equation model (8) proved the correlation between the producer’s knowledge reserve, work experience and production safety accidents. Second, the most important factor affecting production safety accidents and fatalities is unsafe operation, such as not familiar with safety production regulations (9) and violating production regulations (10). In all recent and past production safety accidents, operation error or violation of production regulations is the most common cause. Third, the characteristics of workers are related to production safety accidents. Meta and ratio (11) analysis shows that there is a significant correlation between production accidents and workers’ attention to safe production (12), and the awareness of safe production (13) has a significant impact on safe production. Therefore, being good at learning and managing knowledge plays an important role in promoting safe production (14).

Technical factors. As the technical carrier of production activities, production equipment is an important technical factor causing production safety accidents (15). Generally, the longer the service life of production equipment, the higher the probability of mechanical failure and production accidents (16). For example, technical equipment defects are the root cause of affecting coal mine safety. In addition, the loss of technical knowledge is an important factor leading to major accidents. Some studies believe that knowledge loss caused by lack of new technology, insufficient training and information, and insufficient exchange of technical experience (17) may lead to major accidents. It can be seen from this that technical factors are the biggest factors affecting work accidents (18). Management factors. First, the faster the managers react and handle, the lower the accident losses will be. For example, if positive measures and rapid response measures are taken to prevent accidents, the casualty rate will be significantly reduced (19). Therefore, carrying out sound safety management training and developing advanced safety culture concepts will help to fundamentally improve the safety situation (20). Secondly, poor management is the key factor leading to accidents (21). On the one hand, the safety management ability of production enterprises (22) directly affects the severity of production safety accidents. The analysis results show that the explosion of dangerous goods at the port has complex causal factors, but the management factor plays a leading role in the causal structure of the whole accident (23). On the other hand, the ability to deal with and deal with accidents (24) is also very important to reduce the severity of accidents. Safety management education and training (25) is an important way to remedy the shortcomings of safety management actions (26). Environmental factors. External pressure from government regulators and other stakeholders (27) has an important impact on production safety accidents. The stronger the external supervision pressure, such as regular inspection, making plans or regulations, will help reduce the incidence of production safety accidents (28). The research shows that the regulatory authority is the most influential level in the causal relationship of accidents, and the lack of effective safety supervision mechanism is the core influencing factor of accidents (29). However, some studies have found that strong external supervision may not reduce the incidence of production safety accidents (30).

Therefore, the existing research has basically reached a consensus on the causes of production safety accidents, mainly focusing on individual factors, technical factors, management factors and environmental factors. However, it is worth noting that the existing research on the causes of major production safety accidents has not clearly solved the following problems:

First, in terms of research methods, existing studies mainly explore the causal relationship of major production safety accidents through large sample statistical analysis or single case analysis. However, large sample statistical analysis or single case study (6) cannot distinguish the characteristics of different accidents, covering the unique laws of major production safety accidents. The accident prevention benefit analysis (4) model and ratio (5) were used to analyze the causes of production safety accidents, and the causal relationship between accident severity and different factors was not fully considered. This means that it is necessary to explore the complex causal relationship of major production safety accidents with new research methods. The occurrence mechanism of major production safety accidents may be different from general production safety accidents. Therefore, it is necessary to conduct a more detailed study on the nature and necessary conditions of major production safety accidents.

The study on the interaction between the causes of major production safety accidents needs to be deepened. Existing research usually devoted to analyzing the relationship between a single factor and the consequences of a particular accident. However, they ignore the discussion of the interaction among the influencing factors. The existing studies have not yet revealed the causal relationship between the specific factors that cause major production safety and their combination modes. In fact, production safety accidents are the result of coupling of complex factors (11). A single factor may not be enough to constitute a key factor determining the severity of production safety accidents, but a single factor can be coupled with other factors to jointly affect the severity of accidents. “The linear combination of multiple variables can effectively explain the characteristics and patterns of accidents” (31), “unsafe behaviors, inadequate supervision, technical equipment failures and other factors interact in a complex way, affecting the scale of production safety accidents” (7). In this study, we try to establish a causal coupling theory of production safety accidents that emphasizes the causal relationship.

The impact of external regulatory pressure on the consequences of production safety accidents is uncertain. The risk of production violations and the severity of accidents is obviously high. Strengthening external supervision is essential to reduce production violations and the severity of accidents (29). However, in the absence of production violations, the impact of external supervision on the severity of production safety accidents is not significant. External regulatory pressure sometimes affects the consequences of production accidents, and sometimes does not. Therefore, the complex causal relationship between external regulatory pressure and major production safety accidents has not been answered.

In view of the above problems, this study, from the perspective of configuration analysis, uses 37 production safety incidents and fuzzy set qualitative comparative analysis (fsQCA) to analyze the occurrence path and governance mechanism of major production safety accidents, so as to improve the level of production safety. The structure of the paper is as follows: The second part is the research methods, mainly including the introduction of research design and ideas, case sources and variable design. The third part is the analysis of the empirical results, mainly including the single factor necessity and sufficiency analysis, the combination analysis of sufficient conditions and the generation mechanism of major production safety accidents. Discussions and conclusions are provided in sections 4 and 5.

## 2. Research methods

### 2.1. Research design and ideas

Traditional methods of researching influencing factors focus mainly on quantitative analysis, which makes it difficult to explore the causal relationships underlying complex results. This study employs the qualitative comparative analysis method proposed by Charles, an American sociologist, to analyze the causal relationships underlying complex results. The method of qualitative comparative analysis, based on fuzzy mathematics and set theory and combines quantitative mathematical statistical analysis with qualitative data analysis is a novel research method in social science. Qualitative comparative analysis shows that a specific outcome or output *(Y)* is actually the result of the comprehensive action of several mutually related influencing factors *(X)*. That is, a particular result may be caused by a single influence or by a combination of multiple complex factors. Qualitative comparative analysis thus combines the advantages of traditional quantitative and qualitative research (32) and can explain the path by which multiple complex factors *(X)* influence specific results *(Y) via* an analysis of complex data or qualitative text data. This approach allows qualitative comparative research to transcend the conceptual limitations of traditional quantitative or qualitative research. In qualitative comparative analysis, consistency and coverage are two basic indicators used to measure the reliability of the results obtained through qualitative comparative analysis, both of which are assigned a value ranging between 0 and 1. In general, the closer the values assigned to consistency and coverage are to 1, the more reliable the results. However, this claim is not absolute; a strict definition must be based on the theory and case data in question, and typically, a value higher than 0.75 is regarded as a good result.

The specific operation steps of this method are as follows: First, define the result variables and condition variables of the study, formulate calibration rules, and assign values to variables according to case facts and theoretical knowledge. The membership degree of variables is defined as the fuzzy set between non subordinate relationship (0) and full subordinate relationship (1) (33). Second, a truth table shall be established to present the score combination of condition variables and result variables of each case in detail. Third, the truth table is imported into fsQCA software for calculation, and the relationship between conditions and results is simplified through Boolean algebra to obtain the necessary and sufficient condition combination of results. For more detailed software operation manual, see Ragin, 2017 (34).

For the research questions in this paper, fsQCA has the following adaptability. FsQCA is suitable for analyzing small and medium sample size, and can analyze multi cause concurrent events. Owing to the difficulty to obtain official data related to major production safety accidents, it is impossible to carry out large sample complex statistical research, and regression analysis is not applicable. We collected 37 production safety accidents in China from 2008 to 2022 through authoritative websites. Use fsQCA to reveal the coupling relationship between different factors and reveal the combined path of major production safety accidents.

From the existing literature research, major production safety accidents are a typical causal logic involving many factors. FsQCA can show the coupling relationship between different factors and reveal the generation path and occurrence mechanism of major production safety accidents through combination condition analysis. Therefore, this study adopts the fsQCA method to explore the occurrence mechanism of major production safety accidents. Because it is impossible to measure the cases that do not occur in reality, this study will solve the measurement problem by discussing the generation path and generation mechanism of Major Production Safety Accidents (MPSAs), including major accidents and extraordinarily accidents in this paper, and Ordinary Production Safety Accidents (OPSAs), including ordinary accidents and relatively major accidents in this paper.

### 2.2. Data sources

The choice of the number of cases will affect the research results, and an appropriate number of cases will help to present some results more truly. fsQCA is suitable for the study of medium case size (10–50 cases) and small case size (2–10 cases) (35). In combination with the availability of case data, this study selects a medium number of cases for research.

The sources of production safety accident cases in this study mainly include several aspects. First of all, in order to ensure the authority and integrity of the case, we searched the official website of the Ministry of Emergency Management of the People’s Republic of China for the investigation report of major production safety accidents in 2008–2022, and we collected 10 extraordinarily major accidents. We choose the major production safety investigation report which posted on the security management website^1^ as a supplement. Based on the safety management website, collect 8 recent major production safety accidents, a total of 18 accidents, and establish a test group. Secondly, following the principle of difference of QCA method, it is necessary to expand the differences between cases, find out different ways or combinations of specific results, and combine relatively major accident and ordinary accidents into a set for research. According to the selection rules of qualitative comparative analysis cases, the cases in the control group should account for about half of the total cases. At the same time, the qualitative comparative analysis requires that the cases have a certain degree of homogeneity, so we choosed the cases that are close to the occurrence time of the cases in the test group as the cases in the control group. Finally, 19 OPSAs close to the time of MPSAs were selected from the official website of the Emergency Management Department and the safety management website as the control group.

Based on the above steps, 37 production safety accidents were included in the case database (see Appendix Table A1). Among them, 18 MPSAs (8 major accidents and 10 extraordinarily major accidents) were taken as the research objects, accounting for 48.6% of the total number of cases. The control group consisted of 19 OPSAs (7 ordinary accidents and 12 relatively major accident), accounting for 51.4% of the total case base.

### 2.3. Variable operation and assignment

**Figure 1:**
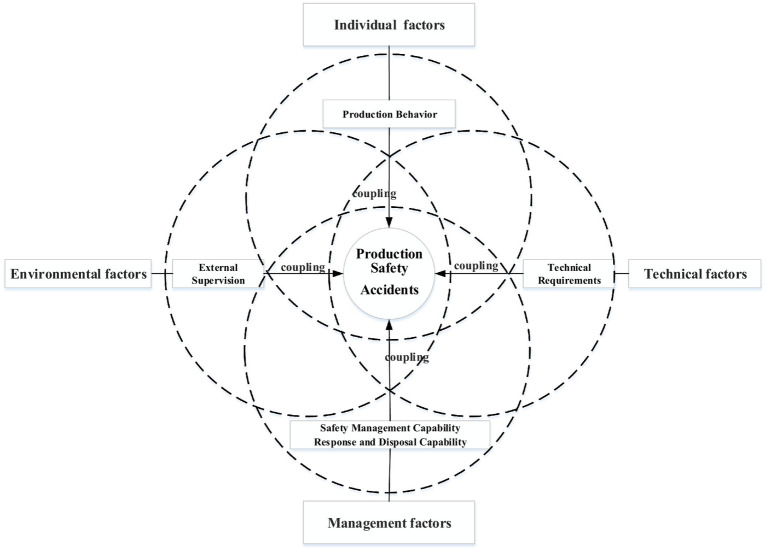
The analysis mode of the configuration effect for major production safety accidents.

The qualitative comparative analysis method requires the selection of variables to meet the requirements of theoretical knowledge and practice. Therefore, the selection of variables follows two principles: (1) theoretical feasibility, that is, it has been proved by existing research to have an important impact on major safety production accidents; (2) Practical feasibility: it has good compatibility with the research case. Research variables include result variables and condition variables. In this study, MPSAs are taken as outcome variables, and the selection process of condition variables is as follows: On the one hand, the main influencing factors in Figure 1 are summarized from existing research results, including individual factors, technical factors, management factors and environmental factors. In many production safety accident investigation reports, external supervision is the key way to prevent production safety accidents. This paper takes external supervision as a sub variable of environmental factors, and analyzes the combination path of external supervision and other three factors leading to the result variable.

On the other hand, the measurement of other conditional variables needs to be realized through sub variables. The variable selection recommended by fsQCA is between 3 and 7 (36), and it is more inclined to select those sub variables that have significant influence on the result variable. Based on this, the condition variables shown in Figure 1. mainly consider the importance, influence and representativeness of sub variables, rather than comprehensiveness. This measurement method is mainly used to find the variables that have the most direct impact on the outcome variables. Based on case facts and theoretical knowledge, and fully considering the operability of variables, Production Behavior (PB), Technical Requirements (TR), Safety Management Capability (SMC), Response and Disposal Capability (RDC), and External Supervision (ES) are selected as conditional variables. The research framework of “Individual – Technology – Management – Environment” is established, as shown in Figure 1. On this basis, the interaction and coupling path between the main influencing factors are analyzed. The arrows in the figure indicate the interaction between the influencing factors. The solid wireframe is a sub variable of influencing factors, which is used to measure the main influencing factors. The raw data is converted to gradient fractions by calibration (37). The assignment process of result variable and condition variable is shown in Table 1. In addition, the qualitative comparative analysis method usually requires the variable value to be between 0 and 1 (38). The numerical value represents the degree of membership of a factor. For example, 0 means no affiliation at all, and 1 means complete affiliation. The values between 0–1 are divided into partial membership and non-partial membership according to the degree difference.

**Table 1:** The operational definition and assignment of variables.

Types	Variables	Sub-variables	Assignment rules	Assignment	Assignment support
Outcome variables	Severity of production safety accidents	–	MPSAs	1	Investigation report facts
			OPSAs	0	
Condition variables	Individual factors	Production behavior	Irregular production and operation errors	1	Investigation report facts
			Production behavior meeting production requirements	0	
	Technical factors	Technical Requirements	The accident subject meets the production technical requirements	1	Query through enterprise information website
			The accident subject does not meet the production technical requirements	0	
		Safety management capability	Meet safety management capability	1	Investigation report facts
			No safety management capability	0	
	Management factors	Response and disposal capability	Time from the occurrence of the accident to the end of the disposal	1	Assign values based on Quartiles
				0.01	
	Environmental factors	External supervision	Number of regulatory policies in the 3 years before the accident	1	Assign values based on quartiles
				0.01	

① According to the severity of the accident, OPSAs, MPSAs are assigned the values of 0 and 1, respectively. ② The value is assigned according to whether there is any production violation or operation error. Yes is 1, and No is 0. ③ According to the accident investigation report, whether the production behavior of the accident subject meets the technical requirements is assigned by two points: 1 for meeting the technical requirements, and 0 for not meeting the technical requirements. ④ According to the investigation report, the expression of safety management ability is assigned by two points. ⑤ The ability of accident response and disposal is largely reflected in the time from the occurrence to the end of the accident. ⑥ As a tool to implement the government’s intentions, policies can represent the government’s regulatory intensity on specific aspects (39). Therefore, the external supervision is based on the number of regulatory policies issued by the local provincial government within 3 years before the accident. The response capacity and external supervision strength are expressed by the fuzzy set of quartiles. The lower quartile (25%) of the data is regarded as a completely independent calibration point, the median (50%) is regarded as an intersection point, and the upper quartile (75%) is regarded as a completely independent calibration point. The fuzzy set is used as a measure of emergency response capability. In addition, in order to avoid cases that are difficult to be classified and excluded due to intersection, the membership value of 0.5 is set as 0.51 (40). To further quantify the difference between the two, set the case frequency to 1, the consistency threshold to 0.8, and the PRI consistency threshold to 0.7 (37). Further calculating the necessity and sufficiency of single factor to measure the importance of condition variables.

## 3. Research result

### 3.1. Single factor necessity and sufficiency analysis

We use fsQCA3.1b software to analyze the necessary and sufficient conditions for major safety production accidents. The operation results are shown in Table 2. In the necessary condition test, the condition variable with consistency greater than 0.9 is considered as a necessary condition for the result variable (41). If the consistency value of the variable is between 0.8 and 09, the variable can be regarded as a sufficient condition for the result. Therefore, small external supervision is a necessary condition for the occurrence of MPSAs. Abnormal production behaviors (such as production violations and operational errors) and insufficient safety management capacities are sufficient conditions for the occurrence of MPSAs. Abnormal production behavior is a necessary condition for OPSAs, and insufficient response and disposal capacity is a sufficient condition for OPSAs. Next, we analyzed the configuration of condition variables to obtain more information about the path of MPSAs.

**Table 2:** The necessity test of a single condition based on the fsQCA method.

Condition variables	Outcome variables	
	Consistency of MPSAs	Consistency of OPSAs
PB	0.522	0.578
~PB	0.877	0.921
TR	0.555	0.421
~TR	0.444	0.578
SMC	0.166	0.315
~SMC	0.833	0.684
RDC	0.443	0.579
~RDC	0.556	0.820
ES	0.498	0.650
~ES	0.905	0.350

### 3.2. Configuration analysis of sufficient conditions

In this study, we use fsQCA3.1b software to analyze the intermediate solution and simple solution of MPSAs. The statistical analysis results are shown in Table 3. In the analysis results, QCA requires that the consistency of the solution should be greater than 0.8, and the coverage should be higher than 0.5. This shows that our results meet the requirements for consistency and coverage. A configuration path is a combination of condition variables that produce a result variable. The results show that there are five paths of MPSAs. The consistency of the five configuration paths is 1, indicating that the path combinations of the five configurations are all MPSAs. The coverage rate is 0.680, indicating that the five interpretation paths can explain 68% of MPSAs. In addition, the core condition and edge condition are distinguished based on the combination of simplified solution and intermediate solution (37). In Table 3, the condition variables appearing in the intermediate solution and the simple solution together are the core variables, which are represented by “▲” in the path. Other condition variables are auxiliary conditions, which have relatively small impact on the result variables, and are represented by “*.” At the same time, we use “–” for missing or non-existent condition variables.

**Table 3:** The realization of the configuration of MPSAs by using fsQCA.

Condition variables	Sub-variables	Conditional configuration of MPSAs				
		Path 1	Path 2	Path 3	Path 4	Path 5
		Technology – Management		Individual – Technology – Management	Individual – Technology – Management – Environment	
Individual factors	Production behavior	–	–	▲	▲	–
Technical factors	Technical requirements	*	▲	▲	–	▲
	Safety management capability	▲	▲	▲	–	*
Management factors	Response and disposal capability	▲	*	*	▲	▲
Environmental factors	External supervision	–	–	–	▲	▲
Original coverage		0.316	0.222	0.204	0.388	0.074
Unique coverage		0.130	0.372	0.188	0.056	0.037
Total consistency		1				
Total coverage		0.68				

* means auxiliary conditions, ▲ means core conditions, and – means conditions do not exist.

On the basis of sufficient condition configuration analysis, we further discuss the characteristics of condition variables of MPSAs. Firstly, the five combination paths conform to the causal relationship of multiple causes and one effect. Different paths are composed of different condition variables, which are characterized by multi factor concurrency. Based on the original coverage, the interpretation ability of different paths to the results is ranked as path 4 > path 1 > path 2 > path 3 > path 5. Secondly, it is generally believed that abnormal production behavior (such as production violations and operation error) is the main cause of MPSAs. For example, Path 3 and Path 4 are caused by abnormal production behavior. However, Path 1, Path 2, and Path 5 indicate that normal production behavior may also lead to MPSAs when there are other harmful factors. Paths 1, 2, and 5 indicate that accidents may also occur if production technology requirements are not met, management capacity is insufficient or external supervision is weak. It is mainly due to the coupling effect of individual factors, technical factors, management factors and environmental factors in the process of occurrence of MPSAs. Thirdly, meeting or failing to meet the condition variables may lead to major production safety accidents. For example, compliance with production technology requirements (Path 1), timely response and disposal (Path 2, Path 3) and strong safety management capability (Path 5) may also lead to MPSAs.

### 3.3. Generation mechanism of MPSAs

According to the conditional variable configuration analysis, the generation mode of MPSAs is summarized and refined. According to five combined paths, three modes of MPSAs are extracted. That is, MPSAs induced by “Technology - Management” coupling (Path 1, Path 2), MPSAs induced by “Individual – Technology – Management “coupling (Path 3), and MPSAs induced by “Individual – Technology – Management – Environment” coupling (Path 4, Path 5).

MPSAs induced by “Technology – Management.” The core conditions here include non-compliance with production technical requirements, insufficient safety management ability and response and disposal ability. This shows that in the absence of abnormal production behavior and effective external supervision, violation of production technical requirements, confusion in production management and insufficient emergency response capacity are the main reasons for MPSAs. The typical accidents induced by the coupling of technology and management factors include the “11.24” cooling tower collapse accident in Fengcheng, Jiangxi Province.

MPSAs induced by “Individual – Technology - Management.” Combination path 3 is a typical case that represents the coupling of individual, technology and management factors to induce MPSAs. The core conditions here include abnormal production behavior, non-compliance with production technology requirements and insufficient safety management capability, and the combination of response and disposal capabilities as marginal conditions. In this kind of MPSAs, although the main body of the accident has a strong emergency response capability, the extremely serious production safety accidents have resulted in a large number of casualties and property losses due to factors such as production violations, operational errors, ignoring production technical requirements, and confusion in the organization’s internal safety management. The typical accident induced by the coupling of individual, technology and management factors is the “8.2” explosion accident in Kunshan, Jiangsu Province.

Major production safety accidents induced by “Individual – Technology – Management – Environment.” The core conditions here include abnormal production behavior, non-compliance with production technology requirements, insufficient response and disposal capacity and weak external supervision. This shows that even though the safety management ability is strong, due to the lack of effective external supervision, producers are likely to violate production and operation rules in pursuit of output, leading to MPSAs. The typical accident induced by the coupling of individual technology management environment factors is the “10.31” gas explosion accident in Yongchuan, Chongqing.

According to the above analysis, it is obvious that there are three modes of MPSAs. First, the coupling mode of “Technology – Management” factors with the core elements of non-compliance with production technology requirements, insufficient safety management ability and response and disposal ability. Second, the coupling mode of “Individual – Technology – Management” factors, which is mainly caused by abnormal production behavior, non-compliance with production technology requirements and insufficient safety management ability. Third, the coupling model of “Individual – Technology – Management – Environment” factors, which is mainly caused by abnormal production behavior, non-compliance with production technology requirements, insufficient response and disposal capacity, and weak external supervision. These three occurrence modes indicate that MPSAs are the result of multi factor coupling.

## 4. Discussion

With the help of qualitative comparative analysis method, this study establishes an “Individual – Technology – Management – Environment” analysis framework to analyze the path and mode of MPSAs. The theoretical contribution of this study to the existing literature is mainly reflected in four aspects.

Aiming at the problem that the existing literature pays little attention to the occurrence mechanism of MPSAs. Our work reveals the facts covered by the large sample statistical research. The necessary conditions for OPSAs and MPSAs are generally different. On the basis of single factor comparative analysis, the differences between the sufficient and necessary conditions of MPSAs and OPSAs are presented in detail. Specifically, under the condition that the production technology requirements are met, minor external supervision, abnormal production behaviors (such as illegal production and operational errors) and insufficient safety management capability are more likely to lead to MPSAs. However, abnormal production behavior, insufficient response and disposal capacity are the main reasons for OPSAs.

Respond to the lack of consideration of the interaction of different factors in MPSAs. Different from traditional large sample statistical research, which pays attention to single factor and ignores interaction between variables, we use Boolean algebra operation of set theory to understand which combination of factors will lead to MPSAs from the perspective of configuration. Based on the coupling analysis framework of “Individual – Technology – Management – Environment” system, a new research idea is proposed. The research results verify the hypothesis of scholars that MPSAs are the result of multiple factors coupling, and give the specific path of MPSAs caused by different factors coupling. Based on the production safety accident cases, this study considers the interaction between the four main factors of production safety accidents and explores the five main paths of production safety accidents. It can better understand the specific path and mode of MPSAs and promote the research from influencing factors to occurrence mechanism. Based on conditional configuration analysis, it is proved that “a single factor can be used as a catalyst or barrier when combined with other factors that affect the severity of damage” (42). The five paths and three modes of MPSAs present the core causes and mechanisms of MPSAs in detail and completely. It helps to formulate production safety management policies and methods and improve production safety management level.

Respond to the dispute about the uncertain impact of external supervision on MPSAs. Based on the analysis results, we agree that external supervision is an important factor affecting MPSAs (28). In the single factor necessity and sufficiency results of MPSAs, the consistency of small external supervision is high (value is 0.905). This result, to some extent, refutes the view that external supervision has no significant impact on the severity of production safety accidents. More importantly, this paper believes that external supervision plays different roles in different combination paths of MPSAs. Paths 1, 2, and 3 prove that the absence or invalidity of external supervision will be difficult to curb abnormal production behavior and increase the severity of the accident (43). Paths 4 and 5 prove that in the presence of external supervision, coupling with other factors may still lead to MPSAs. Therefore, this study responds to the controversy about the uncertain impact of external supervision on MPSAs, and believes that external supervision has an impact on the severity of accidents in different paths. However, it should also be pointed out that the impact of external supervision on the severity of MPSAs depends on the coupling of external supervision and other factors.

It provides a comprehensive analysis framework for analyzing MPSAs. The analysis framework of “Individual – Technology – Management – Environment” coupling not only provides framework support for the research and analysis of the causes and modes of MPSAs, but also provides an expandable framework basis for the future expansion of research on MPSAs. Researchers can develop or revise the framework to produce new knowledge by incorporating more updated production safety accident case data. At the same time, it can also be used in accident research in other fields to provide support for accident research in other fields. Therefore, this is an inclusive and developmental analytical framework.

## 5. Conclusion

In this work, we used fsQCA to study 37 production safety accidents, to define the influencing factors and generation mechanism of MPSAs, and try to answer the three questions mentioned in the introduction. First, what are the causes of MPSAs? It is found that MPSAs are the result of interaction of multiple influencing factors under the coupling framework of “Individual – Technology – Management – Environment.” At the same time, there are some differences between the sufficient and necessary conditions for OPSAs and MPSAs. Among them, small external supervision (single factor consistency is 0.905) is a necessary condition for MPSAs. Abnormal production behavior (single factor consistency is 0.877) and insufficient safety management capability (single factor consistency is 0.833) are sufficient conditions for MPSAs. Abnormal production behavior (single factor consistency is 0.921) is a necessary condition for OPSAs, and insufficient response and disposal capacity (single factor consistency is 0.820) is a sufficient condition for OPSAs. Second, how do the influencing factors of major production safety accidents interact? From the analysis of conditional variable configuration, no matter whether individual, technical, environmental and management factors are met, major production safety accidents have main occurrence modes. Third, what is the path of MPSAs? Based on the research and analysis results, we summarized the five conditional variable combination paths of MPSAs into three paths: “Technology – Management” coupling mode, “Individual – Technology – Management” coupling mode and “Individual – Technology – Management – Environment” coupling mode. Comparing the original coverage of different paths, the technology – management coupling mode is more likely to cause MPSAs. The priority interpretation capability combination based on the original coverage is ranked as Path 4 (0.388) > Path 1 (0.316) > Path 2 (0.222) > Path 3 (0.204) > Path 5 (0.074).

In order to reduce MPSAs, “Technology – Management” coupling mode, “Individual – Technology – Management” coupling mode and “Individual – Technology – Management – Environment” coupling mode are decomposed according to the combination path of necessary conditions and condition variables. First, reduce abnormal production activities and maintain a stable and sustainable external environment. The second is to combine the actual situation of MPSAs, determine the accident path according to the configuration path special diagnosis, and carry out targeted disposal and correction. In the daily production safety management, we should pay attention to maintaining appropriate external supervision, improving the enterprise’s safety management ability, simultaneously cultivating the production safety response and disposal ability, and building a “Individual – Technology – Management – Environment” comprehensive governance system for MPSAs.

In the future, we can try to continue to promote the research from the following four aspects, deepen the theoretical research of major production safety accidents, and enrich the theoretical knowledge of the research of accident path and mechanism. First, researchers can try to expand the scope of case samples to increase the credibility and universality of research conclusions. Alternatively, the configuration analysis method can be combined with the large sample statistical research to establish a more scientific hybrid research paradigm to jointly verify or optimize the research conclusions of this paper. Secondly, researchers can further explore the occurrence path and mode of ordinary accidents, relatively serious accidents, major accidents and major accidents. Distinguish the leading factors of different degrees of safety production accidents, and then establish a hierarchical attribution model that matches the severity of the accident. Provide framework guidance for the prevention and disposal of production safety accidents. Third, from the micro level, production behavior and external supervision can be further subdivided according to the severity of the accident. For example, subdivide the impact of illegal production, operational errors and lack of experience of manufacturers, or study whether different levels of government regulation have a differentiated impact on the severity of accidents. Try to provide governance guidance and theoretical knowledge reference for a specific type of safety production accidents. Finally, the theoretical model of “individual-technology-management-environment” constructed in this paper is an inclusive analysis framework. Therefore, the framework can provide researchers with a framework basis and reference standards to explore the occurrence path and mechanism of other types of accidents, thus enriching the causal model of accident research.

## Data availability statement

The original contributions presented in the study are included in the article/supplementary material, further inquiries can be directed to the corresponding author.

## Author contributions

FZ: methodology, software, investigation, data curation, writing-original draft preparation, writing-review and editing, funding acquisition, and resources. JZ: validation, formal analysis, data curation, writing – review and editing, and supervision. CF: methodology, formal analysis, writing – original draft preparation, writing – review and editing, visualization, funding acquisition, and conceptualization. All authors contributed to the article and approved the submitted version.

## Conflict of interest

The authors declare that the research was conducted in the absence of any commercial or financial relationships that could be construed as a potential conflict of interest.

## Publisher’s note

All claims expressed in this article are solely those of the authors and do not necessarily represent those of their affiliated organizations, or those of the publisher, the editors and the reviewers. Any product that may be evaluated in this article, or claim that may be made by its manufacturer, is not guaranteed or endorsed by the publisher.

## Footnotes

^1^ http://www.safehoo.com/

## Appendix A

**Table A1:** List of selected case database of production accidents that occurred in China between 2008 and 2022.

Num.	Types	Cases	Num.	Types	Cases
1	Extraordinarily major	Jiangsu Xiangshui “3.21” Explosion Accident in 2019	20	Relatively major	Fujian Hongmiaoling “5.17” Explosion Accident in 2022
2	Extraordinarily major	Jiangxi Fengcheng “11.24” Collapse Accident in 2016	21	Relatively major	Zhejiang Tiantai “11.20” Explosion Accident in 2021
3	Extraordinarily major	Chongqing Yongchuan “10.31” Gas Explosion Accident in 2016	22	Relatively major	Hebei Gaoyang “8.17” Gas Explosion Accident in 2021
4	Extraordinarily major	Inner Mongolia Chifeng “12.3” Gas Explosion Accident in 2016	23	Relatively major	Hebei Chicheng “4.7” Explosion Accident in 2021
5	Extraordinarily major	Tianjin Binhai New Area “8.12” Explosion Accident in 2015	24	Relatively major	Shaanxi Yulin “7.15” Water Inrush Accident in 2021
6	Extraordinarily major	Henan Pingdingshan “5.25” Fire Accident in 2015	25	Relatively major	Heilongjiang Anda “12.19” Explosion Accident in 2020
7	Extraordinarily major	Jiangsu Kunshan “8.2” Explosion Accident in 2014	26	Relatively major	Inner Mongolia Ordos “4.30” Tar Burner Explosion Accident in 2020
8	Extraordinarily major	Jilin Tonghua “3.29” Gas Explosion Accident in 2014	27	Relatively major	Hainan Haikou “9.24” Sludge Treatment Explosion Accident in 2020
9	Extraordinarily major	Shandong Baoli “5.20” Explosion Accident in 2013	28	Relatively major	Hubei Zhijiang “2.19” Combustion and Explosion Accident in 2019
10	Extraordinarily major	Yunnan Qujing “11.10” Coal and Gas Outburst Accident in 2011	29	Relatively major	Jiangxi Jiujiang “7.2” Explosion Accident in 2017
11	Major	Guizhou Qianxinan “2.25” Roof Accident in 2022	30	Relatively major	Shanxi Qixian “6.13” Explosion Accident of Low-Temperature Insulated Gas Cylinder in 2016
12	Major	Shanxi Xinzhou “6.10” Water Inrush Accident in 2021	31	Ordinary	Shandong Zibo “10.26” Explosion Accident in 2021
13	Major	Xinjiang Changji “4.10” Water Inrush Accident in 2021	32	Ordinary	Guangdong Yingde “3.3” Deflagration Accident in 2021
14	Major	Guangxi Nandan “10.28” Mine Collapse Accident in 2019	33	Ordinary	Shaanxi Xi’an “11.10” Gas Explosion Accident in 2020
15	Major	Hubei Badong “12.5” Coal and Gas Outburst Accident in 2016	34	Ordinary	Hebei Xuanhua “9.30” Mine Accident in 2020
16	Major	Jilin Baishan “3.6” Coal and Gas Outburst Accident in 2016	35	Ordinary	Hebei Chengde “2.17” Methane Explosion Accident in 2017
17	Major	Heilongjiang Shuangyashan “10.18” Gas Accident in 2013	36	Ordinary	Jiangsu Jiangyin “10.14” Explosion Accident in 2017
18	Major	Qianqiu Coal Mine of Henan “6.5” Rock Burst Accident in 2008	37	Ordinary	Hebei Chengde “8.24” Blasting Accident in 2016
19	Relatively major	Nenan Anyang “1.5” Explosion Accident in 2022			
